# Applying the Unified Theory of Acceptance and Use of Technology to Identify Factors Associated With Intention to Use Teledelivered Supportive Care Among Recently Diagnosed Breast Cancer Survivors During COVID-19 in Hong Kong: Cross-Sectional Survey

**DOI:** 10.2196/51072

**Published:** 2024-06-27

**Authors:** Nelson C Y Yeung, Stephanie T Y Lau, Winnie W S Mak, Cecilia Cheng, Emily Y Y Chan, Judy Y M Siu, Polly S Y Cheung

**Affiliations:** 1 JC School of Public Health and Primary Care The Chinese University of Hong Kong Hong Kong China (Hong Kong); 2 Department of Psychology The Chinese University of Hong Kong Hong Kong China (Hong Kong); 3 Department of Psychology The University of Hong Kong Hong Kong China (Hong Kong); 4 Department of Applied Social Sciences The Hong Kong Polytechnic University Hong Kong China (Hong Kong); 5 Hong Kong Breast Cancer Foundation Hong Kong China (Hong Kong)

**Keywords:** telehealth, tele-delivered supportive cancer care, breast cancer, COVID-19, technology acceptance, UTAUT

## Abstract

**Background:**

Many supportive cancer care (SCC) services were teledelivered during COVID-19, but what facilitates patients’ intentions to use teledelivered SCC is unknown.

**Objective:**

The study aimed to use the unified theory of acceptance and use of technology to investigate the factors associated with the intentions of breast cancer survivors (BCS) in Hong Kong to use various types of teledelivered SCC (including psychosocial care, medical consultation, complementary care, peer support groups). Favorable telehealth-related perceptions (higher performance expectancy, lower effort expectancy, more facilitating conditions, positive social influences), less technological anxiety, and greater fear of COVID-19 were hypothesized to be associated with higher intentions to use teledelivered SCC. Moreover, the associations between telehealth-related perceptions and intentions to use teledelivered SCC were hypothesized to be moderated by education level, such that associations between telehealth-related perceptions and intentions to use teledelivered SCC would be stronger among those with a higher education level.

**Methods:**

A sample of 209 (209/287, 72.8% completion rate) women diagnosed with breast cancer since the start of the COVID-19 outbreak in Hong Kong (ie, January 2020) were recruited from the Hong Kong Breast Cancer Registry to complete a cross-sectional survey between June 2022 and December 2022. Participants’ intentions to use various types of teledelivered SCC (dependent variables), telehealth-related perceptions (independent variables), and sociodemographic variables (eg, education, as a moderator variable) were measured using self-reported, validated measures.

**Results:**

Hierarchical regression analysis results showed that greater confidence using telehealth, performance expectancy (believing telehealth helps with daily tasks), social influence (important others encouraging telehealth use), and facilitating conditions (having resources for telehealth use) were associated with higher intentions to use teledelivered SCC (range: *β*=0.16, *P*=.03 to *β*=0.34, *P*<.001). Moreover, 2-way interactions emerged between education level and 2 of the telehealth perception variables. Education level moderated the associations between (1) performance expectancy and intention to use teledelivered complementary care (*β*=0.34, *P*=.04) and (2) facilitating conditions and intention to use teledelivered peer support groups (*β*=0.36, *P*=.03). The positive associations between those telehealth perceptions and intentions were only significant among those with a higher education level.

**Conclusions:**

The findings of this study implied that enhancing BCS’ skills at using telehealth, BCS’ and their important others’ perceived benefits of telehealth, and providing assistance for telehealth use could increase BCS’ intentions to use teledelivered SCC. For intentions to use specific types of SCC, addressing relevant factors (performance expectancy, facilitating conditions) might be particularly beneficial for those with a higher education level.

## Introduction

### Potential Impacts of COVID-19 on Breast Cancer Care

The COVID-19 pandemic has been an international public health emergency, posing severe threats to lives and health care systems worldwide. In Hong Kong, the implementation of different preventive measures (eg, regulations for social distancing, reprioritization of hospital services) affected the lives of not only the general population but also individuals with chronic diseases. Being one of the most commonly diagnosed cancers in Hong Kong, breast cancer diagnosis and treatment delays occurred during the COVID-19 pandemic [1]. For example, the number of pathologic specimens for the 4 most common cancer regions in Hong Kong (including breast cancer) received by public laboratories and public hospitals for cancer diagnostic services reduced by 15.5% overall in 2020, compared with the prior 3-year average [2]. Another study suggested that breast cancer patients in Hong Kong needed to wait 3 weeks longer for their first specialist consultation during the COVID-19 crisis than before the pandemic [3].

After completion of active treatments, many breast cancer survivors (BCS) still need supportive cancer care (SCC) and rehabilitation services to help with different cancer-related life aspects [4]. In the Netherlands, one-third of 1051 surveyed BCS reported difficulties contacting their general practitioner due to COVID-19 [5]. The COVID-19–related lockdowns in the United States and Germany also disrupted patients’ referrals to cancer survivorship programs [6,7]. To reduce the impact of COVID-19 on cancer care, alternative modes of SCC delivery are therefore important.

### Acceptability of Telehealth for Cancer Patients

Research suggests that COVID-19 might have catalyzed new models of health care (eg, telehealth) [4]. Telehealth is the use of technology to deliver health care, health information, or health education at a distance [8]. Telehealth technologies (including telephone, videoconferencing, and internet-based intervention) can bring services into the patient’s home and help them cope with their illness without the need to be physically present at a hospital or clinic [8]. A recent qualitative study in Australia reported that patients with hematological cancer considered telehealth an acceptable alternative during the pandemic [9]. However, some patients encountered difficulties using teledelivered cancer care services due to a lack of knowledge and skills, plus some preferred to see the doctor visually through a video call over other teledelivered options [9]. Another survey explored the prospect of using telemedicine for follow-up among Australian BCS and found that 70% of respondents had suitable devices to access telehealth but only 15% accepted the postoperation teleconsultation with their surgeon [10]. Given that relevant research is limited in the Hong Kong context, this study examined the level of acceptability of telehealth for BCS to access SCC and its associated factors amid the COVID-19 pandemic.

### Telehealth-Related Perceptions as Determinants of Patients’ Intentions to Use Telehealth for SCC

Different theoretical models have been applied to explore intentions to use telehealth among general healthy populations and patient populations outside the COVID-19 context [11]. Among the models, the unified theory of acceptance and use of technology (UTAUT) is one of the most influential theories to understand people’s acceptance of different types of information technologies including telehealth [11]. According to the UTAUT, performance expectancy (whether the individuals believe using the system would provide benefits), effort expectancy (whether the system is easy to use), social influence (perception of important others’ opinions about using the system), facilitating conditions (organizational and technical infrastructure supporting the use of the system), and technology anxiety (users’ negative emotional states related to learning to use technology [eg, nervousness, fear]) are the important determinants of people’s intentions to use technology [12]. Compared with other traditional behavioral theories (eg, Theory of Planned Behavior, Health Belief Model), the UTAUT seems to have stronger explanatory power for understanding people’s intentions to use telehealth [11].

The model has been applied to people’s use of telehealth in different disease contexts. For example, higher performance expectancy, lower effort expectancy, more favorable social influences, less technology anxiety, and more facilitating factors have been associated with intention to use telehealth among Chinese populations (eg, older individuals in the community, individuals with chronic diseases) [13,14]. Performance expectancy and social influence were associated with higher intention to use telehealth service and treatment among patients with diabetes in Korea [15]. Similarly, among patients with type 2 diabetes in South Africa [16], lower performance expectancy, lower effort expectancy, less social influence, and fewer facilitating conditions explained the generally lower intention to use telehealth services. To the best of our knowledge, research on examining cancer survivors’ intentions to use teledelivered SCC during the COVID-19 pandemic was limited. Therefore, this study aimed to examine how telehealth-related perceptions were associated with intentions to use telehealth for SCC among BCS in Hong Kong during the COVID-19 pandemic.

### Individual Characteristics and Fear of COVID-19 as Potential Determinants of Intentions to Use Telehealth for Supportive Cancer Services Among BCS

In addition to telehealth-related perceptions, patients’ sociodemographic characteristics might also contribute to the acceptability of telehealth [11]. Factors like age, education, possession of smart device(s), the nature of the consultation (routine follow-up versus urgent need for physical examination), and experience with using technology could contribute to the acceptability of telehealth for cancer survivors [17]. Specific to the pandemic situation, recent studies found that fear of COVID-19 transmission was associated with higher intentions to use contact tracing apps among the general population in Germany [18] and telehealth services among cancer patients in the United States [19]. Expecting the same phenomenon to apply to BCS in Hong Kong, we aimed to examine the roles of patients’ individual characteristics (eg, sociodemographic and clinical factors, fear of COVID-19) and prior experience with using technology in intentions to use telehealth for SCC.

### Moderating Role of Education Level

Despite the wide use of the UTUAT to explain people’s intentions to use technology, whether the contribution of the variables in the theory differs based on people’s sociodemographic and individual characteristics has not been extensively examined. Prior studies have generally regarded sociodemographic variables as covariates for intentions or behavior, which fails to unpack the complex ways in which such characteristics might interact with beliefs to determine behavioral intention and actual behaviors (eg, [20-22]). Education level has been suggested as a potential moderator between perceptions about behaviors and intentions to engage in online behaviors. For example, studies measured the intention of individuals to use e-banking based on the UTAUT model in the United Kingdom and Jordon and found that education level had a positive moderating effect on performance expectancy, facilitating conditions [23], and effort expectancy [24]. Another study in Indonesia also found that education level moderated the relationship between effort expectancy and intention to use e-money services [25]. Similar research on the intentions of BCS to use telehealth amid the COVID-19 pandemic was limited. Specifically, the role of education as a moderator between telehealth perceptions and BCS’ intentions to use teledelivered SCC were investigated in this study.

### Purpose of the Study

**Figure 1:**
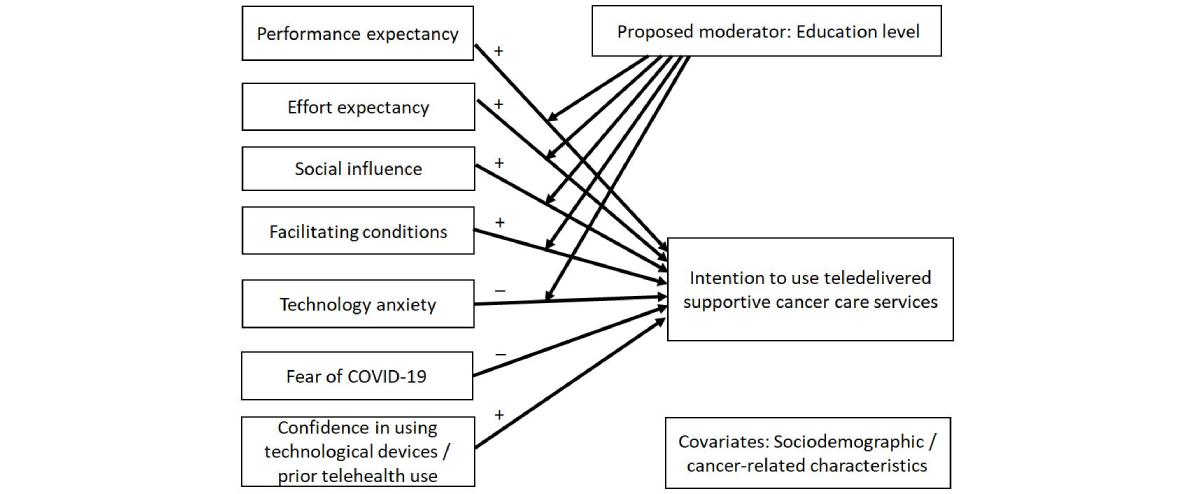
Conceptual model of the study.

This study aimed to examine how telehealth-related perceptions contribute to the intention to use telehealth for cancer care among BCS in Hong Kong during the COVID-19 pandemic (Figure 1). We hypothesized that favorable telehealth-related perceptions (higher performance expectancy, lower effort expectancy, more facilitating conditions, positive social influences), less technological anxiety, and greater fear of COVID-19 would be associated with higher intention to use telehealth for SCC. We also hypothesized that the associations between telehealth-related perceptions and intentions to use teledelivered SCC would be moderated by education level, such that associations between telehealth-related perceptions (higher performance expectancy, lower effort expectancy, more facilitating conditions, positive social influences, less technological anxiety) and intention to use teledelivered SCC would be stronger among those with a higher education level.

## Methods

### Participants and Procedure

A cross-sectional study was conducted. BCS were eligible to participate if they (1) were older than 18 years, (2) had a confirmed diagnosis of Stage 0-III breast cancer since the outbreak of COVID-19 in Hong Kong (January 2020), (3) were receiving active treatment (eg, radiotherapy, chemotherapy), (4) could read Chinese to answer questionnaires and communicate in Cantonese, and (5) were able to provide meaningful informed consent. BCS were excluded if they had (1) a history of any psychiatric disorder, (2) metastatic brain disease, (3) any other type of cancer, or (4) recurrent breast cancer.

Prospective participants were recruited from the Hong Kong Breast Cancer Registry (HKBCR). The HKBCR has been the most comprehensive, representative local data collection and monitoring system for BCS in Hong Kong [26]. Upon approval, BCS who fulfilled the inclusion criteria based on the data in the HKBCR were invited to participate in the study through telephone calls. Of the 943 BCS contacted, 409 were not reachable, 23 were not eligible, and 227 were not interested in the study. With initial verbal consent via phone, those who were eligible and interested in the study (N=287) were asked to complete the cross-sectional survey. Participants received a cover letter explaining the study details, consent form, packet of questionnaires, stamped return envelope, thank you/reminder letter, and replacement packet via mail. After consent, participants completed the survey in the home setting. Telephone calls were used to remind individuals who had not returned the questionnaires. The study was conducted between June 2022 and December 2022 (amid the fifth wave of the COVID-19 pandemic in Hong Kong) [27]. A total of 209 completed surveys were returned (out of 287 sent), yielding a completion rate of 72.8%.

### Ethical Considerations

Ethics approvals were sought from the Joint Chinese University of Hong Kong - New Territories East Cluster Clinical Research Ethics Committee (CREC Ref. 2021.286) and Hong Kong Breast Cancer Foundation. We obtained informed consent before participation in the survey. Upon completion of the survey, participants received supermarket vouchers (worth HK$100; approximately US $12.80) to compensate them for their time. We guaranteed that the identity of the participants would not be revealed.

### Sample Size Planning

The dependent variable was the intention to use teledelivered SCC services. Based on prior studies on the acceptability of telehealth among Chinese populations [28,29], we expected a small to medium overall effect size (*f*^2^=0.10) in the association between telehealth-related perceptions and intentions to use telehealth services in the hierarchical regression analysis. To achieve a statistical power of .80 at α=.05, a minimum of 201 participants were needed (G*Power 3.1.2). The sample size (N=209) achieved via the recruitment strategy was expected to allow the detection of the expected effect size with sufficient statistical power.

### Measures

A written, closed-ended, anonymous, self-administered questionnaire was used in the study. To ensure that the questionnaire was readily comprehensible, a pilot test was conducted among 10 BCS who were eligible for the study. The study questionnaire was finalized based on feedback from the pilot test participants.

### Intention to Use Telehealth for Future Supportive Cancer Services

Participants’ intentions to use telehealth for future supportive cancer services was measured using a SCC service utilization scale [30] that was modified according to the local health care context. The checklist covered different categories of services, including psychological support (6 items; α=.91), medical consultation (5 items; α=.86), integrated or complementary care (6 items; α=.87), and peer support (2 items; α=.83). On a 4-point scale (1, no intention or not applicable; 2, low intention; 3, moderate intention; 4, high intention), participants were asked to indicate their intention to use telehealth for each SCC service (eg, “I intend to use telehealth for ‘psycho-oncology counseling.’”). The scale has been shown to be reliable and valid among Western cancer survivors [30].

### Perceptions About Telehealth for SCC Services

We used 4 subscales (performance expectancy [3 items], effort expectancy [4 items], social influence [3 items], and facilitating conditions [3 items]) to measure participants’ perceived usefulness, perceived ease, social influence, and facilitating conditions, respectively, for using telehealth in cancer care [31]. Sample items include “Using telehealth for cancer care is beneficial to my health.” (α=.83; performance expectancy), “It is easy for me to become skillful at using telehealth for cancer care service.” (α=.87; effort expectancy), ”People whose opinions that I value (eg, my doctors) think I should use telehealth for cancer care services.” (α=.86; social influence), and ”I have the resources necessary to use telehealth for cancer care services.”(α=.90; facilitating conditions). On a 5-point scale (1, strongly disagree; 5, strongly agree), higher mean item scores from the scales indicate higher levels of the corresponding constructs. The Chinese versions of these scales were shown to be reliable and valid among Chinese adults [32].

### Technology Anxiety

A 3-item scale was adapted to measure participants’ technology anxiety while using telehealth services [14]. On a 5-point scale (1, strongly disagree; 5, strongly agree), a higher mean item score indicates a higher level of technology anxiety (eg, “I feel nervous about using telehealth.” α=.91). The Chinese version of the scale was shown to be reliable and valid among Chinese adults [14].

### Fear of COVID-19

The Chinese version of the 7-item Fear of COVID-19 scale was adapted to measure participants’ fear of COVID-19 [33]. On a 5-point scale (1, strongly disagree; 5, strongly agree), a higher mean item score indicates a higher level of COVID-19 fear (eg, “It makes me uncomfortable to think about COVID-19.” α=.88). The scale has been shown to be reliable and valid in the Chinese population [34].

### Clinical and Sociodemographic Characteristics

Participants self-reported their (1) sociodemographic characteristics (eg, age, education level, employment status, marital status), (2) treatment-related variables (eg, surgeries undergone, treatments receiving or undergone, time since last treatment), (3) daily living variables (eg, access to the internet, use of electronic or mobile devices), and (4) breast cancer-related variables (eg, stage at diagnosis, time since diagnosis).

### Cancer Care Experiences During COVID-19

Participants were asked if they had participated in any telehealth online consultation sessions for SCC (including psychological support services, medical support services, integrated and complementary support services, spiritual support services, other support services; no=0, yes=1).

### Statistical Analysis

Descriptive and bivariate Pearson correlation analyses were conducted. Hierarchical regression analyses were also conducted to examine factors associated with intentions to use telehealth for supportive cancer services. The sequence of entering independent variables followed suggestions from prior studies that examined factors associated with people’s health or health behavior outcomes and the interaction effects among those factors (eg, [35,36]). The process usually involves entering important sociodemographic and individual experience variables in the first block (as a statistical control for confounding variables), variables representing major theoretical constructs in the next block(s), and the interaction terms between the proposed moderating variable and the independent variables of interest in the last block. In our study, fear of COVID-19 and the sociodemographic and clinical variables that had significant bivariate correlations with the dependent variables were entered in block 1 of the regression model. Telehealth-related perceptions (ie, performance expectancy, effort expectancy, social influence, facilitating conditions, technology anxiety) were entered into block 2 of the regression model. In the last block, 5 interaction terms between telehealth-related perceptions and education level were entered into the model. To compute the interaction terms, the mean-centered scores of telehealth perceptions and education level (binary: college level versus below college level) were multiplied. All continuous independent variables were centered prior to the analyses. For statistically significant interactions, simple slopes analyses [37] were conducted to examine how the main effects of telehealth perceptions on intentions to use teledelivered SCC varied at different education levels. Those with *P*≤.05 in the final regression model were considered statistically significant. These analyses were performed using SPSS version 26.0.

## Results

### Participant Characteristics

Among the 209 participants, 82 (39.2%) were 50 years or younger, 63 (30.1%) were 51 years to 60 years old, and 62 (29.7%) were at least 61 years old. In addition, of the 209 participants, 91 (43.5%) had a tertiary education, 72 (34.4%) worked full-time, 99 (47.4%) reported a religious affiliation, and 53 (25.4%) had a comorbid chronic illness. Regarding cancer-related characteristics, 10 (4.8%), 60 (28.7%), 86 (41.1%), and 53 (25.4%) of the 209 participants reported being diagnosed with Stage 0, Stage I, Stage II, and Stage III breast cancer, respectively, and 194 (94.3%) had undergone breast cancer surgery. The average time since diagnosis was 16.6 (SD 8.00) months. Regarding internet access, 204 of the 209 participants (97.6%) had a mobile phone with internet access (Table 1).

**Table 1:** Demographic characteristics of the participants (N=209).

Characteristics		Results
**Age (years), n (%)**		
	≤50	82 (39.2)
	51-60	63 (30.1)
	≥61	62 (29.7)
	Refused to answer	2 (1)
Gender (female), n (%)		209 (100)
**Cancer stage, n (%)**		
	Stage 0	10 (4.8)
	Stage 1	60 (28.7)
	Stage 2	86 (41.1)
	Stage 3	53 (25.4)
Time since diagnosis (months), mean (SD)		16.6 (8.0)
Breast cancer surgery, n (%)		197 (94.3)
**Type of breast cancer surgery, n (%)**		
	Lumpectomy	103 (49.3)
	Axillary dissection	126 (60.3)
	Mastectomy	97 (46.4)
	Breast reconstruction	25 (12)
**Treatment, n (%)**		
	Chemotherapy	152 (72.7)
	Radiotherapy	159 (76.1)
	Targeted therapy	60 (28.7)
	Immunotherapy	8 (3.8)
Comorbid chronic illness (yes), n (%)		53 (25.4)
**Educational level, n (%)**		
	Primary education	15 (7.2)
	Secondary education	102 (48.8)
	Tertiary and higher	91 (43.5)
	Refused to answer	1 (0.5)
**Marital status, n (%)**		
	Single	33 (15.9)
	Married	153 (73.6)
	Divorced or widowed	22 (10.6)
**Monthly household income (HK$), n (%)**		
	≥10,000	46 (22)
	10,001-30,000	42 (20.5)
	30,001-50,000	43 (20.6)
	>50,000	35 (16.7)
	Refused to answer	42 (20.1)
Had a religious affiliation, n (%)		99 (47.4)
**Employment status, n (%)**		
	Full-time	72 (34.4)
	Part-time	20 (9.6)
	Retired, housewife, unemployed, or other	114 (54.5)
	Refused to answer	3 (1.4)
Had a mobile phone with internet access, n (%)		204 (97.6)
Had an electronic device with internet access, n (%)		170 (81.8)

### Intentions to Use Teledelivered SCC Services

Participants’ intentions to use different types of teledelivered SCC services are presented in Table 2. Almost all the teledelivered SCC services listed were accepted by most of the participants. The most accepted teledelivered SCC services in different categories were psychooncology counseling (140/209, 67%), nutrition consultation (165/209, 78.9%), movement and exercise activities (146/209, 69.9%), and patient support groups (131/209, 62.6%).

**Table 2:** Acceptability of teledelivered supportive cancer care services among breast cancer patients (N=209).

Teledelivered supportive cancer care services		Reporting moderate or high intention to use, n (%)
**Psychosocial care**		
	Psychotherapy	117 (56)
	Psychological counseling and support	119 (56.9)
	Psychooncology counseling	140 (67)
	Therapist-led group	133 (63.6)
	Cancer prevention and adaption offers for patients and healthy family members	113 (54)
	Family counseling	71 (34)
**Medical consultation**		
	Cancer helpline	130 (62.2)
	Special medical consultation	132 (63.1)
	To get a second opinion about treatment options	128 (61.2)
	Palliative care consultation	129 (61.7)
	Expert consultation	126 (60.3)
	Nutrition consultation	165 (78.9)
	Complementary and alternative medicine (including traditional Chinese medicine) consultation	139 (66.5)
**Complementary care**		
	Movement and exercise activities (eg, yoga, qigong, exercises for pain relief)	146 (69.9)
	Creative therapeutic offers (music and art therapy)	105 (50.2)
	Relaxation, breathing, meditation exercise group sessions	121(57.9)
	Mindfulness exercises	103 (49.2)
	Massage exercises	108 (51.7)
**Peer support groups**		
	Internet forum with peers	95 (45.5)
	Patient support group	131 (62.6)

### Correlations Between Major Variables and Intention to Use Telehealth

The correlation analysis results showed that the participants with a higher education level, prior telehealth experience, and more confidence using technology devices were more likely to report a higher intention to use telehealth (Table 3). Older age was associated with lower intentions to use 3 different types of teledelivered oncology services. Higher levels of performance expectancy, effort expectancy, facilitating conditions, and social influence were associated with higher intentions to use teledelivered oncology services. A higher level of technology anxiety was negatively correlated with intentions to use teledelivered oncology services. Contrary to the hypotheses, fear of COVID-19 was not associated with intentions to use teledelivered oncology services (Table 3). Other demographic characteristics (eg, marital status, *P*=.82; cancer stage, *P*=.83; time since diagnosis, *P*=.18; income, *P*=.10) were not correlated with the intention to use telehealth (data not tabulated).

**Table 3:** Correlations among major independent variables and intentions to use teledelivered supportive cancer care services (N=209).

Independent variables			Intention to use psychosocial teledelivered supportive care		Intention to use teledelivered medical consultations		Intention to use teledelivered complementary cancer care		Intention to use teledelivered peer support groups
**1. Age^a^**									
	*r*	–0.20		–0.17		–0.10		–0.28	
	*P* value	.005		.02		.16		<.001	
**2. Education^b^**									
	*r*	0.22		0.16		0.24		0.32	
	*P* value	.001		.02		<.001		<.001	
**3. Prior telehealth use^c^**									
	*r*	0.29		0.24		0.22		0.32	
	*P* value	<.001		.001		.001		<.001	
**4. Confidence using technological devices**									
	*r*	0.34		0.34		0.31		0.30	
	*P* value	<.001		<.001		<.001		<.001	
**5. Fear of COVID-19**									
	*r*	0.05		0.01		–0.05		0.03	
	*P* value	.47		.92		.47		.65	
**6. Performance expectancy**									
	*r*	0.45		0.39		0.36		0.40	
	*P* value	<.001		<.001		<.001		<.001	
**7. Effort expectancy**									
	*r*	0.37		0.32		0.29		0.32	
	*P* value	<.001		<.001		<.001		<.001	
**8. Facilitating conditions**									
	*r*	0.41		0.34		0.30		0.43	
	*P* value	<.001		<.001		<.001		<.001	
**9. Social influence**									
	*r*	0.30		0.32		0.26		0.22	
	*P* value	<.001		<.001		<.001		.001	
**10. Technology anxiety**									
	*r*	–0.18		–0.18		–0.14		–0.22	
	*P* value	.009		.01		.04		<.001	

^a^≤55 years (0); >55 years (1).

^b^High school or less (0); at least college (1).

^c^No (0); Yes (1).

### Hierarchical Regression Analysis

Given that the independent variables were moderately correlated, the independent variables were checked for multicollinearity in the regression analysis. None of the variables had a variance inflation factor ≥5, which indicated the absence of multicollinearity problems.

In block 1, the background variables explained 16.4%, 14.9%, 13.4%, and 20.2% of the variance in the intentions to use teledelivered psychosocial care, medical consultation, complementary care, and peer support groups, respectively. Specifically, a higher education level was associated with higher intentions to use teledelivered complementary care and peer support groups, and greater confidence with using technological devices was associated with higher intentions to use all 4 types of teledelivered SCC services. Prior telehealth use was associated with greater intentions to use teledelivered medical consultation and peer support groups (Tables 4 and 5).

**Table 4:** Hierarchical regression analyses to explain intentions to use telehealth services (N=209).

Steps			Intentions to use teledelivered supportive cancer care																				
			Psychosocial care^a^												Medical consultation^b^								
			*β*		*P* value		ΔR^2^			*P* value			*β*				*P* value		ΔR^2^			*P* value	
**Step 1: Background variables**								0.164			<.001									0.149			<.001
	Age^c^	–0.06		.67								–0.03				.83							
	Education^d^	0.27		.06								0.11				.46							
	Prior telehealth use^e^	0.27		.08								0.38				.01							
	Confidence using technological devices	0.28		<.001								0.29				<.001							
	Fear of COVID-19	0.12		.08								0.05				.46							
**Step 2: Telehealth-related perceptions**								0.159			<.001									0.126			<.001
	Age^c^	–0.02		.89								–0.02				.92							
	Education^d^	0.12		.39								0.02				.91							
	Prior telehealth use^e^	0.19		.19								0.34				.02							
	Confidence using technological devices	0.20		.01								0.23				<.001							
	Fear of COVID-19	0.06		.35								–0.01				.86							
	Performance expectancy	0.34		<.001								0.26				<.001							
	Effort expectancy	–0.07		.47								–0.08				.44							
	Facilitating conditions	0.20		.02								0.12				.19							
	Social influence	0.08		.30								0.16				.03							
	Technology anxiety	0.08		.28								0.06				.41							
**Step 3: Interaction terms**								0.013			.58									0.006			.90
	Age^c^	–0.03		.81								–0.01				.96							
	Education^d^	0.13		.36								0.04				.77							
	Prior telehealth use^e^	0.17		.25								0.32				.03							
	Confidence using technological devices	0.20		.01								0.22				.01							
	Fear of COVID-19	0.07		.30								0.00				.99							
	Performance expectancy	0.30		.01								0.24				.03							
	Effort expectancy	–0.07		.56								–0.05				.68							
	Facilitating conditions	0.19		.09								0.17				.14							
	Social influence	0.09		.35								0.17				.09							
	Technology anxiety	–0.03		.73								0.05				.64							
	Performance expectancy × education	0.07		.67								0.08				.62							
	Effort expectancy × education	0.02		.90								–0.05				.79							
	Facilitating conditions × education	0.03		.85								–0.14				.44							
	Social influence × education	–0.03		.82								–0.03				.83							
	Technology anxiety × education	0.26		.07								0.02				.89							

^a^Total R^2^: 0.336.

^b^Total R^2^: 0.281.

^c^≤55 years (0); >55 years (1).

^d^High school or less (0); at least college (1).

^e^No (0); Yes (1).

**Table 5:** Hierarchical regression analyses to explain intentions to use telehealth services (N=209).

Step		Intentions to use tele-delivered supportive cancer care													
		Complementary care^a^								Peer support groups^b^					
		*β*	*P* value	ΔR^2^		*P* value		*β*			*P* value	ΔR^2^		*P* value	
**Step 1: Background variables**					0.134		<.001						0.202		<.001
	Age^c^	0.17	.26					–0.26			.08				
	Education^d^	0.36	.01					0.45			.002				
	Prior telehealth use^e^	0.21	.19					0.38			.01				
	Confidence using technological devices	0.27	<.001					0.16			.03				
	Fear of COVID-19	0.01	.87					0.10			.12				
**Step 2: Telehealth-related perceptions**					0.096		<.001						0.122		<.001
	Age^c^	0.18	.23					–0.19			.17				
	Education^d^	0.28	.05					0.32			.02				
	Prior telehealth use^e^	0.17	.26					0.30			.04				
	Confidence using technological devices	0.23	.01					0.08			.28				
	Fear of COVID-19	–0.05	.50					0.08			.22				
	Performance expectancy	0.25	.002					0.30			<.001				
	Effort expectancy	–0.05	.64					–0.17			.08				
	Facilitating conditions	0.07	.44					0.26			.002				
	Social influence	0.13	.09					0.03			.70				
	Technology anxiety	0.06	.41					–0.02			.77				
**Step 3: Interaction terms**					0.031		.16						0.029		.13
	Age^c^	0.20	.17					–0.21			.13				
	Education^d^	0.34	.02					0.32			.02				
	Prior telehealth use^e^	0.13	.40					0.31			.03				
	Confidence using technological devices	0.22	.01					0.07			.38				
	Fear of COVID-19	–0.02	.79					0.08			.24				
	Performance expectancy	0.12	.30					0.25			.02				
	Effort expectancy	–0.00	.10					–0.05			.67				
	Facilitating conditions	0.17	.17					0.11			.32				
	Social influence	0.15	.14					0.05			.62				
	Technology anxiety	0.01	.94					–0.11			.23				
	Performance expectancy × education	0.34	.04					0.05			.77				
	Effort expectancy × education	–0.10	.63					–0.29			.13				
	Facilitating conditions × education	–0.27	.13					0.36			.03				
	Social influence × education	–0.09	.56					0.01			.92				
	Technology anxiety × education	0.09	56					0s.22			.12				

^a^Total R^2^: 0.261.

^b^Total R^2^: 0.353.

^c^≤55 years (0); >55 years (1).

^d^High school or less (0); at least college (1).

^e^No (0); Yes (1).

In block 2, telehealth-related perceptions explained an additional 15.9%, 12.6%, 9.6%, and 12.2% of the variance in intentions to use teledelivered psychosocial care, medical consultation, complementary care, and peer support groups, respectively. Specifically, performance expectancy was associated with intentions to use all 4 types of teledelivered SCC services (Tables 4 and 5). More facilitating conditions were associated with higher intentions to use teledelivered psychosocial care and peer support groups. Greater social influence was associated with higher intentions to use teledelivered medical consultation (*β*=0.16, *P*=.03; Tables 4 and 5).

**Figure 2:**
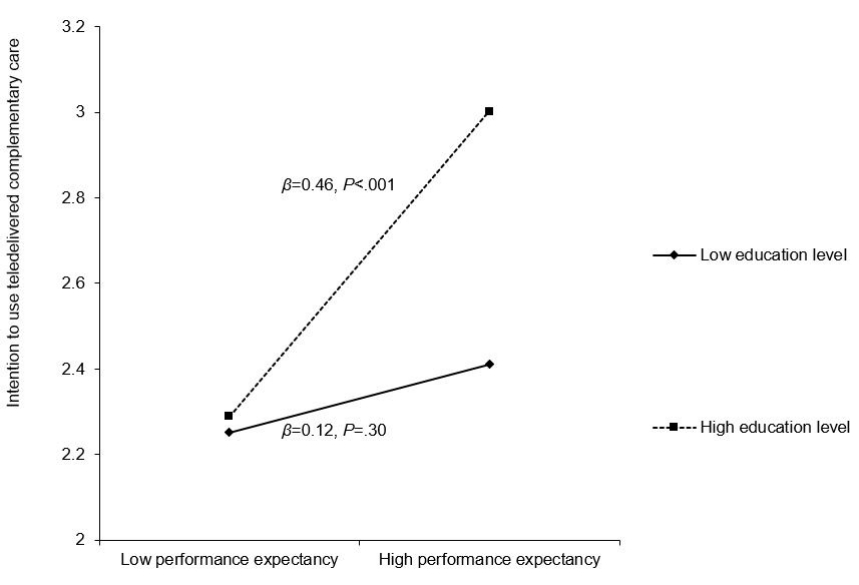
Relationship between performance expectancy and intention to use teledelivered complementary care by education level.

**Figure 3:**
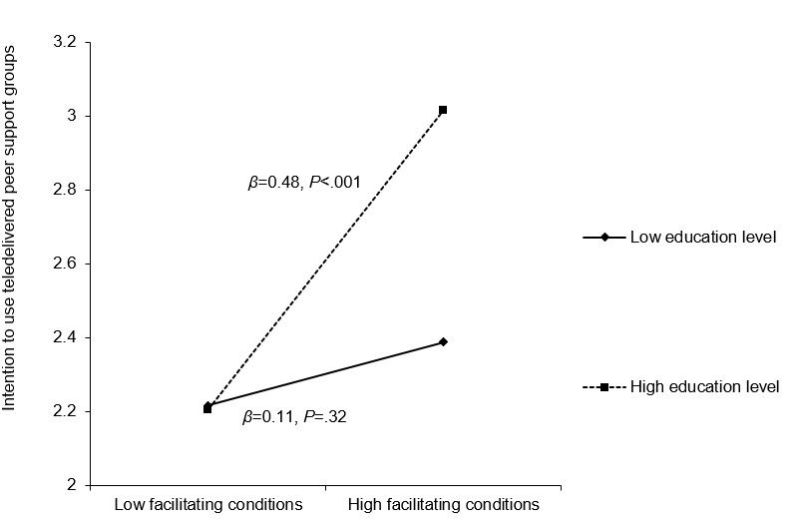
Relationship between facilitating conditions and intention to use teledelivered peer support groups.

In block 3, 5 interaction terms between education level and telehealth-related perceptions were entered; 2 significant interactions emerged. Specifically, there was an interaction between education level and performance expectancy when explaining the intention to use teledelivered complementary care (*β*=0.34, *P*=.04). In addition, there was an interaction between education level and facilitating conditions when explaining the intention to use teledelivered peer support groups (*β*=0.36, *P*=.03). Simple slopes analysis results indicated that the association between performance expectancy and intention to use teledelivered complementary care was only significant among those with a higher education level (*β*=0.46, *P*<.001) but not among those with a lower education level (*β*=0.12, *P*=.30; Figure 2). Similarly, the association between social influence and intention to use teledelivered peer support groups was only significant among those with a higher education level (*β*=0.48, *P*<.001) but not among those with a lower education level (*β*=0.11, *P*=.32; Figure 3). Overall, the models explained 26.1% to 35.3% of the variance in the intentions to use different types of teledelivered SCC services (Tables 4 and 5).

## Discussion

### Principal Findings

This study examined how sociodemographic and clinical factors and telehealth-related perceptions contributed to the intentions to use telehealth for SCC among BCS in Hong Kong during the COVID-19 pandemic. It is noteworthy that most of the participants reported moderate-to-high intentions to use different types of teledelivered SCC services. The most accepted teledelivered SCC services in different categories were psychooncology counseling (67%), nutrition consultation (78.9%), movement and exercise activities (69.9%), and patient support groups (62.6%). We found that greater confidence in telehealth use, performance expectancy (believing telehealth helps with daily tasks), social influence (important others encouraging telehealth use), and facilitating conditions (having resources for telehealth use) were associated with higher intentions to use teledelivered SCC. Our findings were comparable to those of a study in Singapore amid the COVID-19 pandemic [38] that showed that general acceptance of telemedicine by patients with cancer was around 60%. Perceptions that telemedicine could improve health care access and the availability of necessary resources for telemedicine were associated with higher acceptance among those patients [38].

### Sociodemographic Factors, Fear of COVID-19, and Intention to Use Teledelivered SCC

In our regression analyses, education level, prior telehealth use, and confidence using technological devices were associated with the use of telehealth services. Our findings were consistent with findings from patient populations in Western countries supporting that people with mobile device access, who were confident using technological devices, and with prior telehealth experience were more likely to use teledelivered SCC [38-40]. The facilitating roles of those variables seem to be culturally and geographically universal. To increase patients’ intentions to use teledelivered SCC, it might be important to provide education and training on how to use technology and telehealth services, which could help increase confidence with using these tools and make it easier for patients to access care.

Consistent with a population-based study in the United States during the COVID-19 pandemic [41], household income was not a significant contributor to intentions to use teledelivered SCC in our study. However, the findings should be interpreted with caution, as a high proportion of participants (20.1%) refused to report their household income. Household income has been associated with other important sociodemographic factors (eg, education, ownership of mobile devices, internet access) that were associated with cancer survivors’ intentions to adopt telehealth before and during the COVID-19 pandemic [42,43]. Given that 97.6% of our participants possessed a mobile phone with internet access, the unique contribution of household income on intention to use telehealth might become less apparent.

Despite a significant bivariate correlation between age and intention to use teledelivered SCC, age did not emerge as a significant contributor in the regression analyses beyond the influence of other potential contributors. These findings imply that other individual characteristics (eg, confidence using technological devices) played a stronger role in the intentions of BCS to use teledelivered SCC. Moreover, it is important to note that Hong Kong has a very high internet coverage rate at the household level (96.1%) and a very high smartphone ownership rate (99.8% and 90.7% among individuals aged 45-64 years or ≥65 years, respectively) [44], which could influence the acceptability of and perceptions toward telehealth services. The generalizability of our findings to other countries with different internet use patterns should also be interpreted with caution [45].

Fear of COVID-19 did not emerge as a significant contributor to the intention to use teledelivered SCC services in our sample, which was contrary to the findings of prior studies in the United States [19] and Germany [46]. However, our findings seem to be in line with those of An and colleagues [47] who showed that anxiety about COVID-19 was not associated with telehealth acceptance among individuals with chronic disease in South Korea. A potential reason for the discrepancies in the findings could be related to the focus of the measurements. The Fear of COVID-19 scale used in this study primarily measures participants’ affective responses and anxiety symptoms toward cues related to COVID-19, but it might not capture individuals’ perceptions of the threat of contracting COVID-19 at different occasions (eg, hospital and clinic settings, crowded places). Such concerns have been reflected in studies among BCS [48]. Future studies might elucidate how patients’ specific COVID-19 worries and concerns contribute to their intentions to use telehealth services.

### Telehealth-Related Perceptions and Intentions to Use Teledelivered SCC

In the correlation analyses, all the measured telehealth-related perceptions were significantly correlated with the intentions to use SCC. However, in the regression analyses, the relative importance of the perception variables on intentions to use SCC was apparent. Specifically, only performance expectancy was associated with the intention of using all types of the measured teledelivered SCC. Similar findings have also been reported regarding the prediction of the acceptance of cancer patients in the Netherlands to use a virtual assistant in health care settings [49] and the acceptance of using a digital cardiac rehabilitation tool among patients with ischemic heart disease in Germany [50]. The findings imply that highlighting the benefits of teledelivered SCC on daily life for BCS tends to increase their intentions to use such services.

On the other hand, social influence was associated with the intention to use teledelivered medical consultation. In the Chinese culture, coping with cancer is largely a family issue, such that opinions of family members are important in patients’ treatment decision-making [51]. Given that it might also be easier for family members who do not live together to participate in medical consultations, family members might tend to welcome the option to have such consultations teledelivered. That might be the reason why social influence had a relatively strong contribution to the intention of BCS to use teledelivered medical consultations (but not other SCC services).

Furthermore, facilitating conditions were associated with the intention to use psychosocial care services and peer support groups (but not other types of SCC). It is noteworthy that psychological care and peer support group services are not commonly utilized among local BCS [25]. The dynamics in psychological counseling and peer support groups involve more disclosure of personal challenges and distress, which might be incongruent with the cultural preference of not bringing up negative emotions to maintain social harmony [33]. It might be possible for local BCS to believe that they need a certain level of knowledge and informational resources (facilitating conditions) to understand what to expect in teledelivered psychosocial care and peer support groups before enrolling in those services.

Although our findings suggested that effort expectancy and technology anxiety contributed less significantly to intentions to use teledelivered SCC, it is still noteworthy that facilitators and barriers are likely to differ across different cultural contexts and by types of telemedicine service [52]. Future research should investigate how those factors jointly contribute to the acceptability of teledelivered SCC services for BCS.

### Telehealth Perceptions and Intentions to Use Teledelivered SCC: Education Level as a Moderator

We found that education level moderated the interaction between (1) between performance expectancy and intention to use teledelivered complementary care and (2) facilitating conditions and intention to use teledelivered peer support groups. From the perspective of the UTAUT model, performance expectancy (ie, degree to which the individual believes that using the technology will help them better cope with daily life or be more effective) was found to be associated with higher intentions to use teledelivered complementary care (including creative therapies, relaxation, and mindfulness exercises) only among those with a higher education level. It is also noteworthy that similar patterns of findings were also apparent in other aspects of technology use. Education level moderated the positive associations between technology use perceptions (performance expectancy, facilitating conditions) and people’s intentions to use mobile banking services in Jordan [23].

Our findings suggested that just highlighting performance expectancy might not be sufficient to significantly increase intentions to use teledelivered complementary care among those with a lower education level. A basic understanding of those complementary care options might be important. Given that those with higher levels of education may be more likely to have better awareness of the potential benefits of those complementary therapies for oncology care [53], the facilitating role of performance expectancy in the intention of BCS to use teledelivered complementary care could be strengthened by a higher education level.

Similarly, we found that facilitating conditions were associated with higher intentions to use teledelivered peer support groups only among those with a higher education level. Facilitating conditions refer to people’s perceptions about whether the necessary resources and support are available to use the technology effectively. It is important to note that peer support groups generally involve mutual interactions and sharing with other cancer survivors, which could also be subject to challenges such as confrontation involving others’ suffering, divergent information needs, conflicts in group dynamics, and challenges with sustainability [54]. Individuals with higher levels of education may be more comfortable using teledelivered services to interact with other patients with similar (stressful) experiences plus have more resources to deal with the potentially negative experiences in the support group context (eg, worsened health of peers in the group, appraising information about their illness, and therapy options shared in the support groups). These reasons might explain why the facilitating role of facilitating conditions in the intention of BCS to use teledelivered peer support group was only apparent among those with a higher education level.

### Limitations

This study was subject to several limitations. First, this study used a cross-sectional design, which might not highlight the causal relationship among the variables. Cancer survivors’ expectations and motivations for teledelivered cancer care may also change over time. Future studies could use longitudinal designs to better understand the temporal relationships among the variables and their future use of teledelivered care services. Second, to allow more systematic recruitment of recently diagnosed BCS (since the COVID-19 outbreak in Hong Kong), we recruited BCS through local cancer registries. Even though the HKBCR is the most comprehensive registry for BCS in Hong Kong, it is noteworthy that not everyone in the total BCS population was covered due to the HKBCR’s voluntary enrollment system. Based on the Hong Kong Cancer Registry data [55] and HKBCR [56] for individuals with BCS aged 18 years to 70 years, the age group distributions were as follows: 40% (<50 years), 33% (50-59 years), 27% (60-70 years). Similarly, in our sample, the age group distributions were as follows: 39.2% (<50 years), 30.1% (51-60 years), and 29.7% (≥61 years). Our sample was highly comparable in terms of the age distribution of the local BCS. However, the generalizability of the findings to BCS in other regions or countries with different health care systems and to survivors of other cancer types might be limited. Third, the studied variables only explained a moderate proportion of variance in the intentions of BCS to use teledelivered SCC. Other factors might be at play. Research has found that other telehealth-related perceptions (eg, privacy concerns), the specific characteristics of different teledelivered services (eg, expected durations and schedules of the services, the necessity to use cameras for the services, group- and individual-based delivery), and contextual factors (eg, severity of the pandemic situation, availability of specific types of teledelivered care services) could be important determinants for those intentions [18,57]. Consideration of those variables might further improve the explanatory power of the regression model.

### Implications

The COVID-19 pandemic impacted cancer service utilization among cancer patients worldwide. Telehealth can be a new service model for SCC services, especially after the experience of the COVID-19 pandemic. The use of telehealth for SCC not only provides flexibility for services in hospitals and cancer clinics but also potentially improves cancer survivors’ well-being. Recent reviews and trials have found that teledelivered interventions facilitate positive physical and psychological health impacts on cancer survivors [58-60]. Therefore, identifying the potential determinants for people’s intentions to use telehealth for SCC could facilitate the proposal of novel service models.

This was one of the first attempts to examine how telehealth-related perceptions, sociodemographic and clinical characteristics, and cancer care service utilization experiences during COVID-19 contributed to the intention of BCS to use telehealth for SCC during the COVID-19 pandemic in Hong Kong. It is essential for health care providers to be knowledgeable about specific factors facilitating the intention to use telehealth, so that patients’ needs and cancer care preferences can be met, especially for the response to a potential pandemic of an emerging infectious disease in the future.

Researchers have started to advocate for a patient-centered approach to address patients’ facilitators and barriers to using telehealth. By fitting telehealth into the overall patient journey and treatment plan and applying inclusive design principles, the needs of the most vulnerable populations who may not be engaging with telehealth owing to their age, education level, socioeconomic status, technology skills, and experiences could be better addressed [40]. Our findings imply that enhancing BCS’ skills for using telehealth, improving BCS’ and their important others’ perceived benefits of telehealth, and providing assistance for telehealth use could increase BCS’ intentions to use teledelivered SCC. For intentions to use specific types of SCC (eg, complementary care and peer support groups), addressing relevant factors (performance expectancy, facilitating conditions) might be particularly beneficial for those with a higher education level.

## Abbreviations

BCS: breast cancer survivors
HKBCR: Hong Kong Breast Cancer Registry
SCC: supportive cancer care
UTAUT: unified theory of acceptance and use of technology

## Data Availability

Research data will be shared upon request.

## References

[1] Koczwara B (2020). Cancer survivorship care at the time of the COVID-19 pandemic. Med J Aust.

[2] Vardhanabhuti V, Ng K (2021). Differential impact of COVID-19 on cancer diagnostic services based on body regions: a public facility-based study in Hong Kong. Int J Radiat Oncol Biol Phys.

[3] Co THM, Au-Yeung K, Kwong A (2020). Breast cancer presentation, diagnosis and outcomes during COVID-19 pandemic - a single center case control study in Hong Kong.

[4] Uscher J, Alkon C, DePolo J, Leitenberger A (2023). Special Report: COVID-19's Impact on Breast Cancer Care. Breastcancer.org.

[5] Bargon C, Batenburg M, van Stam L, van der Molen D, van Dam I, van der Leij F, Baas I, Ernst M, Maarse W, Vermulst N, Schoenmaeckers E, van Dalen T, Bijlsma R, Young-Afat D, Doeksen A, Verkooijen H (2020). The impact of the COVID-19 pandemic on quality of life, physical and psychosocial wellbeing in breast cancer patients – a prospective, multicenter cohort study. European Journal of Cancer.

[6] Oppong B, Lustberg M, Nolan T, Relation T, Park K, Healy E, Trance A, Klemanski DL (2023). Utilization of cancer survivorship services during the COVID-19 pandemic in a tertiary referral center. J Cancer Surviv.

[7] Dinkel A, Goerling U, Hönig K, Karger A, Maatouk I, Petermann-Meyer A, Senf B, Woellert K, Wünsch A, Zimmermann T, Schulz-Kindermann F (2021). Psychooncological care for patients with cancer during 12 months of the Covid-19 pandemic: Views and experiences of senior psychooncologists at German Comprehensive Cancer Centers. Psychooncology.

[8] Larson J, Rosen A, Wilson F (2018). The effect of telehealth interventions on quality of life of cancer patients: a systematic review and meta-analysis. Telemed J E Health.

[9] Zomerdijk N, Jongenelis M, Short C, Smith A, Turner J, Huntley K (2021). Prevalence and correlates of psychological distress, unmet supportive care needs, and fear of cancer recurrence among haematological cancer patients during the COVID-19 pandemic. Support Care Cancer.

[10] Noble N, Mackenzie L, Carey M, Proietto A, Sanson-Fisher R, Walker G, Silcock J (2019). Cross-sectional survey to inform the development of a telehealth support model: a feasibility study for women undergoing breast cancer surgery. Pilot Feasibility Stud.

[11] Harst L, Lantzsch H, Scheibe M (2019). Theories predicting end-user acceptance of telemedicine use: systematic review. J Med Internet Res.

[12] Dwivedi Y, Rana N, Jeyaraj A, Clement M, Williams M (2017). Re-examining the unified theory of acceptance and use of technology (UTAUT): towards a revised theoretical model. Inf Syst Front.

[13] Zhou ML, Zhao L, Kong N, Campy KS, Qu S, Wang S (2019). Factors influencing behavior intentions to telehealth by Chinese elderly: An extended TAM model. Int J Med Inform.

[14] Tsai J, Cheng M, Tsai H, Hung S, Chen Y (2019). Acceptance and resistance of telehealth: The perspective of dual-factor concepts in technology adoption. International Journal of Information Management.

[15] Rho M, Kim H, Chung K, Choi I (2014). Factors influencing the acceptance of telemedicine for diabetes management. Cluster Comput.

[16] Petersen F, Jacobs M, Pather S, Hattingh M, Matthee M, Smuts H, Pappas I, Dwivedi YK, Mäntymäki M (2020). Barriers for User Acceptance of Mobile Health Applications for Diabetic Patients: Applying the UTAUT Model. Responsible Design, Implementation and Use of Information and Communication Technology. I3E 2020. Lecture Notes in Computer Science(), vol 12067.

[17] Cho Y, Zhang H, Harris M, Gong Y, Smith E, Jiang Y (2021). Acceptance and use of home-based electronic symptom self-reporting systems in patients with cancer: systematic review. J Med Internet Res.

[18] Tomczyk S, Barth S, Schmidt S, Muehlan H (2021). Utilizing health behavior change and technology acceptance models to predict the adoption of COVID-19 contact tracing apps: cross-sectional survey study. J Med Internet Res.

[19] Caston N, Lawhon V, Smith K, Gallagher K, Angove R, Anderson E, Balch A, Azuero A, Huang CHS, Rocque GB (2021). Examining the association among fear of COVID-19, psychological distress, and delays in cancer care. Cancer Med.

[20] Venkatesh V, Morris M, Davis G, Davis F (2003). User acceptance of information technology: toward a unified view. MIS Quarterly.

[21] Kim S, Kim S (2020). Analysis of the impact of health beliefs and resource factors on preventive behaviors against the COVID-19 pandemic. Int J Environ Res Public Health.

[22] Perez L, Elder J, Haughton J, Martinez M, Arredondo E (2018). Socio-demographic moderators of associations between psychological factors and Latinas' breast cancer screening behaviors. J Immigr Minor Health.

[23] Al Mashagba FF, Nassar MO (2012). Modified UTAUT model to study the factors affecting the adoption of mobile banking in Jordan. International Journal of Sciences: Basic and Applied Research (IJSBAR).

[24] Al-Qeisi KI (2009). Analyzing the use of UTAUT model in explaining an online behaviour: Internet banking adoption. Brunel University Research Archive.

[25] Giri RRW, Apriliani D, Sofia A (2019). Behavioral intention analysis on e-money services in Indonesia: using the modified UTAUT model. Proceedings of the 1st International Conference on Economics, Business, Entrepreneurship, and Finance (ICEBEF 2018).

[26] (2019). Hong Kong Breast Cancer Registry Report No. 11 (Issue 2019). Hong Kong Breast Cancer Foundation.

[27] (2023). Archives of Latest situation of cases of COVID-19. Centre for Health Protection of the Department of Health.

[28] Okediji P, Salako O, Fatiregun O (2017). Pattern and predictors of unmet supportive care needs in cancer patients. Cureus.

[29] Košir U, Loades M, Wild J, Wiedemann M, Krajnc A, Roškar S, Bowes L (2020). The impact of COVID-19 on the cancer care of adolescents and young adults and their well-being: Results from an online survey conducted in the early stages of the pandemic. Cancer.

[30] Sarkar S, Sautier L, Schilling G, Bokemeyer C, Koch U, Mehnert A (2015). Anxiety and fear of cancer recurrence and its association with supportive care needs and health-care service utilization in cancer patients. J Cancer Surviv.

[31] Hoque R, Sorwar G (2017). Understanding factors influencing the adoption of mHealth by the elderly: An extension of the UTAUT model. Int J Med Inform.

[32] Zhang Y, Liu C, Luo S, Xie Y, Liu F, Li X, Zhou Z (2019). Factors influencing patients' intentions to use diabetes management apps based on an extended unified theory of acceptance and use of technology model: web-based survey. J Med Internet Res.

[33] Chi X, Chen S, Chen Y, Chen D, Yu Q, Guo T, Cao Q, Zheng X, Huang S, Hossain MM, Stubbs B, Yeung A, Zou L (2022). Psychometric evaluation of the Fear of COVID-19 Scale among Chinese population. Int J Ment Health Addict.

[34] Au A, Lam W, Tsang J, Yau T, Soong I, Yeo W, Suen J, Ho WM, Wong K, Kwong A, Suen D, Sze WK, Ng A, Girgis A, Fielding R (2013). Supportive care needs in Hong Kong Chinese women confronting advanced breast cancer. Psychooncology.

[35] Yeung N, Zhang Y, Ji L, Lu G, Lu Q (2020). Finding the silver linings: Psychosocial correlates of posttraumatic growth among husbands of Chinese breast cancer survivors. Psychooncology.

[36] Ronquillo CE, Dahinten VS, Bungay V, Currie LM (2023). Differing effects of implementation leadership characteristics on nurses' use of mHealth technologies in clinical practice: cross-sectional survey study. JMIR Nurs.

[37] Aiken LS, West SG (1991). Multiple regression: Testing and interpreting interactions.

[38] Chan ZY, Lim CF, Leow JL, Chium FY, Lim SW, Tong CHM, Zhou JJX, Tsi MMY, Tan RYC, Chew LST (2022). Using the technology acceptance model to examine acceptance of telemedicine by cancer patients in an ambulatory care setting. Proceedings of Singapore Healthcare.

[39] Reed M, Huang J, Graetz I, Lee C, Muelly E, Kennedy C, Kim E (2020). Patient Characteristics Associated With Choosing a Telemedicine Visit vs Office Visit With the Same Primary Care Clinicians. JAMA Netw Open.

[40] Jacob C, Sezgin E, Sanchez-Vazquez A, Ivory C (2022). Sociotechnical factors affecting patients' adoption of mobile health tools: systematic literature review and narrative synthesis. JMIR Mhealth Uhealth.

[41] Jaffe D, Lee L, Huynh S, Haskell T (2020). Health inequalities in the use of telehealth in the United States in the lens of COVID-19. Popul Health Manag.

[42] Warinner C, Hayirli T, Bergmark R, Sethi R, Rettig E (2021). Patterns of technology use among patients with head and neck cancer and implications for telehealth. OTO Open.

[43] Darcourt J, Aparicio K, Dorsey P, Ensor J, Zsigmond E, Wong S, Ezeana CF, Puppala M, Heyne KE, Geyer CA, Phillips RA, Schwartz RL, Chang JC (2021). Analysis of the implementation of telehealth visits for care of patients with cancer in Houston during the COVID-19 pandemic. JCO Oncology Practice.

[44] (2023). Thematic Household Survey Report No. 77. The Government of the Hong Kong Special Administrative Region.

[45] Jewett P, Vogel R, Ghebre R, Hui J, Parsons H, Rao A, Sagaram S, Blaes AH (2022). Telehealth in cancer care during COVID-19: disparities by age, race/ethnicity, and residential status. J Cancer Surviv.

[46] Tomczyk S (2022). Appsolutely secure? Psychometric properties of the German version of an app information privacy concerns measure during COVID-19. Front Psychol.

[47] An M, You S, Park R, Lee S (2021). Using an extended technology acceptance model to understand the factors influencing telehealth utilization after flattening the COVID-19 curve in South Korea: cross-sectional survey study. JMIR Med Inform.

[48] Seven M, Bagcivan G, Pasalak S, Oz G, Aydin Y, Selcukbiricik F (2021). Experiences of breast cancer survivors during the COVID-19 pandemic: a qualitative study. Support Care Cancer.

[49] van Bussel MJP, Odekerken-Schröder GJ, Ou C, Swart R, Jacobs M (2022). Analyzing the determinants to accept a virtual assistant and use cases among cancer patients: a mixed methods study. BMC Health Serv Res.

[50] Bäuerle A, Mallien C, Rassaf T, Jahre L, Rammos C, Skoda EM, Teufel M, Lortz J (2023). Determining the acceptance of digital cardiac rehabilitation and its influencing factors among patients affected by cardiac diseases. J Cardiovasc Dev Dis.

[51] Liu J, Mok E, Wong T (2005). Perceptions of supportive communication in Chinese patients with cancer: experiences and expectations. J Adv Nurs.

[52] Pang N, Lau J, Fong SY, Wong CYH, Tan KK (2022). Telemedicine acceptance among older adult patients with cancer: scoping review. J Med Internet Res.

[53] Jones E, Nissen L, McCarthy A, Steadman K, Windsor C (2019). Exploring the use of complementary and alternative medicine in cancer patients. Integr Cancer Ther.

[54] Jablotschkin M, Binkowski L, Markovits Hoopii R, Weis J (2022). Benefits and challenges of cancer peer support groups: A systematic review of qualitative studies. Eur J Cancer Care (Engl).

[55] Hong Kong Cancer Registry, Hospital Authority (2023). Female breast cancer in 2021. Hong Kong Cancer Registry.

[56] Hong Kong Breast Cancer Foundation (2023). Hong Kong Breast Cancer Registry Report No. 15. Research Publications.

[57] Gajarawala S, Pelkowski J (2021). Telehealth benefits and barriers. J Nurse Pract.

[58] Siew L, Teo N, Ang W, Lau Y (2023). Social media-based interventions for patients with cancer: a meta-analysis and meta-regression of randomised controlled trials. J Cancer Surviv.

[59] Ebrahimabadi M, Rafiei F, Nejat N (2021). Can tele-nursing affect the supportive care needs of patients with cancer undergoing chemotherapy? A randomized controlled trial follow-up study. Support Care Cancer.

[60] Gyawali B, Bowman M, Sharpe I, Jalink M, Srivastava S, Wijeratne D (2023). A systematic review of eHealth technologies for breast cancer supportive care. Cancer Treat Rev.

